# MAKING OLD BONES: QUALITY IMPROVEMENT IN OSTEOPOROSIS MANAGEMENT IN A GERIATRIC PRIMARY CARE CLINIC

**DOI:** 10.1093/geroni/igz038.3535

**Published:** 2019-11-08

**Authors:** Meredith Gilliam, Sabrina Vereen

**Affiliations:** University of North Carolina, Chapel Hill, North Carolina, United States

## Abstract

Osteoporotic fractures and their sequelae are a leading cause of morbidity and mortality in older adults. In the United States, nearly 50% of white women and 20% of black women and white men will suffer a fragility fracture in his or her lifetime. Osteoporosis medications reduce the risk of major fragility fracture by 31-62%, but numerous care gaps exist, including screening rates as low as 1-47% and treatment rates as low as 16-30% even after a fracture has already occurred. From January to August 2019, we conducted a multi-faceted quality improvement project at a university hospital-based geriatric primary care clinic, with a goal of improving our rates of osteoporosis screening and treatment. We designed and tested electronic health record-based registries of eligible patients, and developed patient outreach workflows and physician “inreach” workflows. We piloted a bone health clinic. While we did not meaningfully affect the rate of osteoporosis screening, our efforts resulted in an increase in treatment of osteoporosis from 49% to 53%. Documentation of osteoporosis decision making among eligible patients improved from 66% to 80%. In our clinic, ongoing barriers to evidence-based osteoporosis management include competition for time with other medical issues, patient mistrust of medications, and the complexity of decision making around osteoporosis in older adults with polypharmacy and limited life expectancy. Future work must balance the broad application of treatment guidelines via population health tools with the need to individualize treatment decisions for each patient’s overall health and goals of care.

